# *DLX5* expression is monoallelic and *Dlx5* is up-regulated in the *Mecp2*-null frontal cortex

**DOI:** 10.1111/j.1582-4934.2008.00377.x

**Published:** 2008-06-05

**Authors:** Masaru Miyano, Shin-ichi Horike, Shutao Cai, Mitsuo Oshimura, Terumi Kohwi-Shigematsu

**Affiliations:** a Life Sciences Division, Lawrence Berkeley National Laboratory, University of CaliforniaBerkeley, CA, USA; b Frontier Science Organization, Institute for Gene Research, Kanazawa UniversityKanazawa, Japan; c Division of Molecular Genetics and Biofunction, Department of Biomedical Science, Graduate School of Medicine, Tottori University, YonagoTottori, Japan

To the Editor

There has been a controversy as to whether DLX5 is imprinted in human brain and lymphoblastoid cell lines (LCLs), and whether expression of *Dlx5* and *Dlx6* (*Dlx5/6* exist as a bigene cluster [1]), is dysregulated in the *Mecp2*-null mouse frontal cortex.

In the September issue of *Am J Hum Genet.* (Vol 81: 492–506, 2007) [2], Schüle *et al.* published a paper entitled ‘*DLX5* and *DLX6* expression is bi-allelic and not modulated by *Mecp2* deficiency.’ This study concluded that *DLX5* was not imprinted in normal human LCLs nor in human brain, contradicting the conclusions of Okita *et al.* (*Genomics 81*:556–559, 2003) [3] and Horike *et al.* (*Nature Genetics* 37:31–40, 2005) [4]. Schüle *et al.* also claimed that expression of *Dlx5* and *Dlx6* in mouse brain varies greatly among individual mice and is not necessarily up-regulated in the frontal cortex of *Mecp2*-null mice. These findings directly contradict those of Horike *et al.* We have now repeated and verified our previous experiments that are relevant to the points raised by Schüle *et al.* Further, we have also expanded our analyses to determine why Schüle *et al.*, were not able to reproduce our findings. Here are our findings in response to their assertions.

(1) Schüle *et al.* presented quantitative RT-PCR (qRT-PCR) data obtained from frontal cortex tissue samples of male wild-type and *Mecp2*-null mice from several litters. They reported that the expression levels of *Dlx5*, *Dlx6* and the imprinted gene *Peg3* were all highly variable among individual mice, independent of genotype (0.5–3.5-fold difference relative to a single wild-type mouse selected among several litters). Schüle *et al.* therefore concluded that the findings of Horike *et al.*– that there is a 2-fold increase in expression of *Dlx5* and *Dlx6* in *Mecp2*-null mice compared to wild type littermates – were the result of ‘biological noise.’

**Fig. 1:**
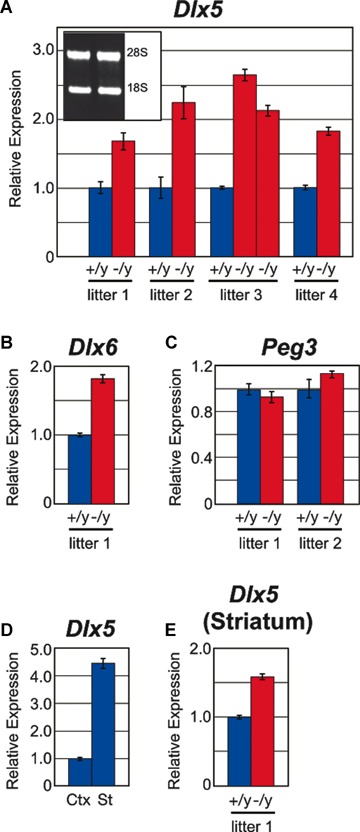
Upregulation of *Dlx5*/*Dlx6*, but not *Peg3*, in the frontal cortex of *Mecp2*-null mice. qRT-PCR results for (**A**) *Dlx5*, (**B**) *Dlx6*, and (**C**) *Peg3* in the frontal cortex of *Mecp2*+/y (blue) and *Mecp2*-/y (red) littermate mice, relative to *Gapdh*. The absence of genomic DNA in the cDNA samples was confirmed. Inset in (**A**) shows electrophoresis of RNA used in qRT-PCR analysis. (**D**) qRT-PCR results for *Dlx5* in the frontal cortex (ctx) and striatum (st) of wild-type mouse brain. (**E**) qRT-PCR results on *Dlx5* in the striatum of *Mecp2*+/y (blue) and *Mecp2*-/y (red) littermate mice. Error bars indicate the S.E.M. Primers sequences for *Dlx5*: 5′-CAGAACGCGCGGAGTTG-3′ and 5′-CCA-GATTTTCACCTGTGTTTGC-3′, *Dlx6*: 5′-TGGCTGCTTCCTTAGGACTGA-3′ and 5′-CTTAGAGCGCTTATTCTGAAACCA-3′, and for *Peg3*: 5′-CCTAT-GAGTATGGGCCCTCCTA-3′ and 5′-CATTCGTACAGTGGGATGTGCTT-3′.

Considering that these genes are tightly regulated during development, we were surprised at their observations that their expression was completely random even among wild type animals from the same litter. We repeated these experiments using three sets of littermates of *Mecp2*-null and wild-type mice (42 or 51 days old) from an independent breeding colony of *Mecp2*-heterozygous mice (generated by Adrian Bird's laboratory) [5] kindly provided by Masayuki Itoh (National Institute of Neuroscience, Japan), who carefully bred the *Mecp2*±heterozygous mice to retain the original phenotype. In all five *Mecp2*-null mice examined using *Gapdh* as a control, we were able to reproduce the ∼2-fold (1.7–2.6-fold) increase in expression of *Dlx5* in *Mecp2*-null mice, compared with wild-type mice (Fig. 1A), just as originally reported by Horike *et al.* We similarly verified up-regulation of *Dlx6* in the same brain subregions of the *Mecp2*-null mice tested (representative data are shown in Fig. 1B). We further examined expression levels of *Peg3*, which Schüle *et al.* also report as having high variability in expression. In contrast to their findings, we observed consistent expression levels with a small standard error of the mean (S.E.M.) between mice of the same genotype and saw similar levels in the frontal cortex between wild-type and *Mecp2*-null mice (Fig. 1C). Expression levels of genes in brains are best compared among littermates, since mice from different litters often show some variability in gene expression. Nonetheless, in our study, difference in expression levels of the control *Gapdh* typically do not exceed more than 20%, even among mice from different litters. Therefore, our results consistently show non-random expression of *Dlx5* and *Dlx6* relative to *Gapdh* among littermates, accurately reflecting the genotype of individual mice within each litter. In the frontal cortex of *Mecp2*-null mice, *Dlx5* and *Dlx6* are moderately up-regulated compared to wild-type mice, while there is no change in expression for *Peg3*.

The differences between our results and those of Schüle *et al.* likely arose from differences in reproducibility in dissection of the frontal cortex and in the purity/quality of RNA; RNA degradation can dramatically alter qRT-PCR results. Because *Dlx5* is expressed at high levels in striatum (greater than 4-fold compared to the frontal cortex (Fig. 1D)), we took great care to ensure that neighbouring striatum (or other tissues) did not contaminate the frontal cortex sample by isolating a narrow region corresponding to the primary and secondary motor cortex (M1 and M2). We also isolated striatal tissue and found *Dlx5* was again moderately up-regulated in this region in *Mecp2*-null mice.

One caution is that after more than 2 years of breeding of *Mecp2* heterozygous mice to ensure a higher chance of survival of *Mecp2*-null(-/y) pups in our laboratory, (either by selecting female mice that are better caretakers, or using a surrogate mother) we found that the phenotype of many *Mecp2*-null(-/y) male mice became milder, with a much extended life span and delayed onset of disease. *Mecp2*-null(-/y) males produced by such mouse lines often show a less than 2-fold increase in *Dlx5* and *Dlx6* (typically a 1.2- to at most 1.5-fold increase compared to control mice). This decline in the fold increase in these mice might be attributed to changes in epigenetic status at these gene loci, and we are currently studying this effect.

(2) Another major point of controversy is that Schüle *et al.* conclude that *DLX5* expression is biallelic in human LCLs and human brains, whereas Horike *et al.*[4] and Okita *et al.*[3] independently conclude that it is imprinted in human LCLs, and Okita *et al.* previously found that it is monoallelically expressed in human brains as well. A total of 18 LCL samples were examined between the two groups (Horike *et al.* and Okita *et al.*), among which 16 showed monoallelic expression. In all eight LCL samples for which the parental origin of *DLX5* was known, *DLX5* was expressed from the maternal allele [3].

**Fig. 2:**
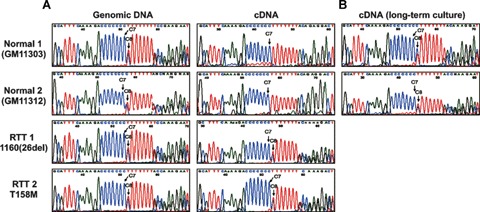
Monoallelic *DLX5* expression in normal individuals and biallelic *DLX5* expression in two individuals with Rett syndrome (RTT). The C7 or C8 mononucleotide repeat polymorphism in the 3′ UTR of *DLX5* is shown. Sequence traces of genomic DNA amplified by PCR and those from the RT-PCR products for *DLX5* are shown for LCLs from normal individuals and individuals with RTT. See Methods section in Horike *et al.*[4]. **A**) LCL cell samples were prepared after minimum passages without additional freezing and thawing after purchased from Coriell Cell Repositories. **B**) The same normal LCLs samples used in **A**) were subjected to several additional passages and one-time freeze/thawing before use.

Schüle *et al.* have examined four human LCLs, and observed only one sample with monoallelic expression (Control 1 in Fig. 2 of Schüle *et al.*), while they observed biallelic expression in all the rest. One important point that needs to be raised here is that if *DLX5* expression were truly biallelic in nature, one cannot expect to obtain data consistent with monoallelic expression, especially after many cycles of amplification. In contrast, if it were monoallelic in nature, one may still obtain sequencing data that could be misinterpreted as biallelic. There are at least two reasons for this: (1) the expression level of *DLX5* is low in human LCLs and requires many PCR cycles, and (2) human LCLs are unstable and can become biallelic under non-optimal culture conditions (more passages than recommended and non-optimal freezing/thawing conditions). Therefore, even if a gene is predominantly expressed from one parental allele, a low-level expression from the other parental allele may still be amplified resulting in sequencing data suggesting biallelic expression for many LCLs. Therefore, the quality of both the LCLs and the RNA are absolutely critical in obtaining reliable data. We repeated the imprinting analyses, using two newly purchased normal human LCLs from Coriell Cell Repositories with minimum prior passages and processed them exactly according to the supplier's instructions. We again observed that *DLX5* was monoallelically expressed in these normal human LCLs. Based on the clean, non-overlapping sequencing data of cDNA derived solely from one parental allele, and the equal contribution of sequences from genomic DNA of both parental alleles (reflected by equal intensity C and T peaks at the point where the sequence mismatch occurs between paternal and material alleles), it is apparent that *Dlx5* is monoallelically expressed in normal LCLs (Fig. 2). We have reproduced the same quality of data (Fig. 2A) as those published previously by Horike *et al.*

To show that LCLs are prone to losing the imprinting status at the *DLX5* locus, we deliberately continued culturing these same cells under non-optimal conditions by several additional passages. After one freeze/thaw cycle, these cells grew at a much slower rate, and *DLX5* expression became biallelic for both cell lines (Fig. 2B). Therefore, we always used freshly purchased LCLs from the supplier with minimum passages to obtain a sufficient number of cells for all experiments involving LCLs. In contrast to normal LCLs, LCLs from two individuals with Rett syndrome (RTT), expanded with a minimum number of passages, exhibited biallelic expression of *DLX5* as reported by Horike *et al.*, and the data were successfully reproduced in Fig. 2A.

In the Schüle *et al.* paper, it is difficult to draw definitive conclusions regarding the mono- or biallelic expression of *DLX5* based on Fig. 2 of Schule *et al.*[2] (for human LCLs) and 3 of Schule *et al.*[2] (for human brain) because of the high level of background sequence noise in multiple cDNA and genomic DNA samples, and the unequal C and T signal intensities at the mismatch for some genomic DNA samples. In mouse brain, *Dlx5* is mostly biallelic [4, 6]. However, in the human brain, Okita *et al.* from Oshimura's lab showed clean sequencing data in all three brain samples, indicating a mostly monoallelic nature of *DLX5* expression. Again, such data could not have been obtained unless transcripts are derived mainly from one allele.

(3) Schüle *et al.* used somatic-cell hybrid lines to show that human *DLX5* and *DLX6* were expressed from both parental alleles. However, the particular cell lines they used have an inherent problem that make them unsuitable for imprinting analyses: individual cell lines have variable copies of multiple human chromosomes. One cell line has lost chromosome 7 that contains *DLX5*/*DLX6*. Furthermore, they all contain an additional active X chromosome, where *Mecp2* is located. We do not know how cells respond to multiple copies of chromosomes especially at the level of gene expression; dysregulation of dosage-sensitive expression of genes may cause unpredictable outcomes. Furthermore, in their experiments, important positive controls were missing, *viz.*, verification of the imprinted status of bona fide imprinted genes located on the human chromosome 7, such as *PEG10* and *PEG1/MEST*. M. Oshimura and colleagues, on the other hand, established a series of human monochromosomal hybrids retaining a single chromosome of defined parental origin [7]. This in vitroassay system is suitable for imprinting analysis and has led to the identification of several imprinted genes [8–10], including *DLX5*, and the data were verified with human LCLs [3]. Collectively, the experiments of Schüle *et al.* using a somatic hybrid cell system do not support their claim that *DLX5* is not imprinted.

We have carefully analyzed the study of Schüle *et al.*, repeating many of their and our own experiments, and stand by our original conclusion that *DLX5* is imprinted in human LCLs, and that this imprinting is lost in some but not all patients with RTT. Furthermore, we stand by our conclusion that chromatin looping defects occur in the brains of *Mecp2*-null mice. Our chromatin immunoprecipitation (ChIP) studies identified *Mecp2* binding sites in the *Dlx5*-*Dlx6* region. To verify the results, ChIP experiments have to be performed carefully with all proper controls. Our data, showing *Mecp2* binding to the *Dlx5*-*Dlx6* locus in mouse brain, were validated by a recent ChIP-chip study in a human neuronal cell line [11].
